# Insomnia among vocational college students in China: Status and influencing factors

**DOI:** 10.4102/sajpsychiatry.v32i0.2595

**Published:** 2026-02-03

**Authors:** Chunying Chang, Jiabao Zhai, Die Hu, Yanan Zheng

**Affiliations:** 1 Jiangxi College of Applied Technology, Ganzhou, China; 2 Department of AIDS and STD Control and Prevention, Shaoxing Center for Disease Control and Prevention, Shaoxing, China; 3 Department of Nursing, Xuzhou Renci Hospital, Xuzhou, China; 4 Department of Psychiatry, Gannan Medical University, Ganzhou, China

**Keywords:** vocational college students, insomnia, sleep disturbance, influencing factors, status

## Abstract

**Background:**

Sleep-related problems, particularly insomnia and sleep disturbances, have become increasingly prevalent. However, few studies have specifically explored these issues among vocational college students.

**Aim:**

To investigate the prevalence and influencing factors of insomnia and its relationship with sleep disturbances in Chinese vocational college students.

**Setting:**

Two vocational colleges in Ganzhou, Jiangxi Province, China.

**Methods:**

A cross-sectional survey was conducted in April 2025 using cluster sampling. A total of 1,993 students completed the Athens Insomnia Scale (AIS), the Espie Sleep Disturbance Questionnaire (SDQ) and a self-designed questionnaire.

**Results:**

In all, 48.02% of students reported insomnia symptoms in the past month. Higher insomnia severity was significantly associated with three SDQ dimensions: sleep restlessness/agitation (*t* = 7.466, *p* < 0.001), mental overactivity (*t* = 9.670, *p* < 0.001) and preoccupation with insomnia consequences (*t* = 9.509, *p* < 0.001). Other significant factors included being female (*t* = −3.582, *p* < 0.001), being a freshman (*t* = −5.782, *p* < 0.001), dissatisfaction with their academic major (*t* = −4.731, *p* < 0.001), alcohol use (*t* = 2.654, *p* = 0.007) and engaging in fewer than 7 h per week of extracurricular study or reading (*t* = −2.328, *p* = 0.020).

**Conclusion:**

Insomnia in vocational college students is influenced by multiple factors and is strongly linked to sleep disturbances.

**Contribution:**

This study provides empirical evidence on insomnia in this population and offers insights to guide targeted interventions in higher education settings.

## Introduction

Sleep is a vital physiological activity for humans, and good sleep quality is directly linked to both physical and mental health.^1,2^ College students, who are currently undergoing critical stages of physical and psychological development, often experience poor sleep quality^3,4^ because of multiple factors such as academic pressure, career planning, postgraduate entrance exams, complex interpersonal relationships, smartphone gaming,^5^ short-form videos^6^ and more. Studies have shown that 20% – 40% of university students suffer from varying degrees of sleep problems.^7,8^ Vocational college students represent a distinct subgroup within the broader college population. Compared with undergraduates, they differ in educational duration, training models, course intensity and career development trajectories. These differences contribute to issues such as smartphone dependence, internet addiction, interpersonal difficulties, low self-confidence and employment concerns, which can adversely affect their mental health.^9,10,11^ Among these problems, sleep-related issues – particularly insomnia and sleep disturbance – have become increasingly prominent in recent years.^12^ However, few studies have specifically focused on the current status, characteristics and influencing factors of these sleep disorders in the vocational college student population. A deeper understanding of these problems is essential for improving the mental health and academic functioning of this group.

In China, studies on sleep problems have predominantly used the Pittsburgh Sleep Quality Index (PSQI),^13,14^ which mainly assesses subjective sleep quality and duration. Nevertheless, this tool lacks sensitivity in identifying issues such as insomnia and sleep disturbance, and its focus on medication use makes it less suitable for younger populations like Chinese university students. Insomnia is a common sleep disorder across various populations, typically manifested as persistent difficulty falling asleep or poor sleep quality.^15^ Sleep disturbance, on the other hand, involves symptoms such as preoccupation with the consequences of insomnia and sleep restlessness or agitation before or during sleep, often accompanied by psychological problems like anxiety.^16^ With increasing academic burdens and the widespread use of digital media – including online gaming and other digital media – behaviour such as staying up late and circadian rhythm disruption has become increasingly common among university students, aggravating sleep disturbance.^17,18^ Whether emotional and other associated issues resulting from alterations in sleep rhythms impact sleep quality, worsen insomnia and exacerbate other sleep problems warrants further investigation. Therefore, investigating the current status of insomnia and its relationship with sleep disturbance among vocational college students and exploring potential influencing factors is of great significance for conducting targeted sleep hygiene education, maintaining the physical and mental health of vocational students and cultivating outstanding applied talents with sound health. We hypothesise that insomnia is positively associated with sleep disturbances among vocational college students.

To better understand the relationship between insomnia and sleep disturbance among vocational college students and to evaluate the applicability of relevant sleep questionnaires in this specific population in China, our research team conducted a survey in April 2025. Using cluster sampling, our study recruited 1993 vocational college students from two institutions – Jiangxi College of Applied Technology and Jiangxi Environmental Engineering Vocational College. The findings are reported as follows.

## Research methods and design

### Subjects

In April 2025, a cluster sampling method was used to select 2086 vocational college students from 85 administrative classes across two grades (freshman and sophomore) at Jiangxi College of Applied Technology and Jiangxi Environmental Engineering Vocational College, covering major academic disciplines at both institutions. To ensure data quality and maximise the response rate, members of the research team – who had previously taught the selected student cohorts and were familiar with their class routines – coordinated the distribution and onsite collection of questionnaires during class breaks. A total of 2086 questionnaires were distributed, 2063 were returned (response rate: 98.90%), and 1993 were valid (validity rate: 95.54%). Inclusion criteria: informed consent. Exclusion criteria: individuals with psychiatric disorders (e.g. depression, anxiety, schizophrenia) or neurological diseases; those currently taking psychotropic medications or other drugs that may affect sleep (e.g. hypnotics, antidepressants, stimulants); individuals with diagnosed chronic conditions that could impact sleep (e.g. asthma, diabetes, chronic pain); and those unable to complete the questionnaire fully or with clearly abnormal responses were excluded.

### Instruments

#### Athens Insomnia Scale

The Athens Insomnia Scale (AIS), developed by Soldatos et al., was used to assess insomnia among vocational college students. This scale is based on the diagnostic criteria for insomnia in the International Classification of Diseases (ICD-10) and evaluates the severity of insomnia symptoms over the past month. The scale contains 8 items assessing sleep onset delay, night awakenings, early morning awakenings, total sleep duration, sleep quality, negative emotional impact, daytime dysfunction and daytime sleepiness. Each item is scored on a 4-point scale (0 = no problem to 3 = severe problem), and the total score is the sum of all items, with higher scores indicating more severe insomnia. A total score ≥ 6 suggests the presence of insomnia and the need for medical or psychological intervention.^16^ In this study, the Cronbach’s α of the scale was 0.87.

#### Espie Sleep Disturbance Questionnaire

The Espie Sleep Disturbance Questionnaire, developed by Espie et al. and localised by Lu Lin et al., was used to assess sleep disturbance among vocational college students. Compared with commonly used sleep quality scales such as the Pittsburgh Sleep Quality Index (PSQI), the SDQ focuses more on participants’ experiences with sleep problems and their perceptions and attitudes toward the causes of insomnia.^19^ It consists of 12 items covering four dimensions: sleep restlessness or agitation, mental overactivity, preoccupation with the consequences of insomnia and insufficient sleep preparation. Each item is rated on a 5-point Likert scale.

Higher scores indicate greater severity of subjective sleep disturbance in the corresponding dimension or overall.^20^ In this study, the Cronbach’s α for the SDQ was 0.90.

#### Self-designed demographic and behavioural questionnaire

Based on relevant studies on factors affecting sleep quality among Chinese college students,^4,9,21,22,23^ a self-designed questionnaire was developed to collect information on gender, age, academic year, major, satisfaction with current major (satisfied or dissatisfied), smoking (yes or no), alcohol consumption (yes/no), bedtime smartphone use (yes or no), weekly physical activity (≥ 150 min or < 150 min), weekly time spent gaming or watching videos on phones/computers (≥ 14 h or < 14 h) and weekly extracurricular learning or reading time (≥ 7 h or < 7 h). Given the behavioural differences between vocational students and the general adult population, smoking was defined as smoking daily for at least six consecutive months,^24^ and drinking was defined as consuming alcohol at least once per week.^25^ Time spent on gaming or watching videos via mobile phone or computer was evaluated using a threshold of 2 h per day.^26^ Learning or reading was defined as spending at least 1 h per day on additional study or reading after completing regular classroom learning (e.g. during evening self-study sessions). According to the recommendations of the World Health Organization (WHO), adults should engage in 150–300 min of moderate-intensity aerobic exercise per week,^27^ and previous studies have shown that such exercise can help improve sleep quality.^28^ However, considering the limitations of cross-sectional studies and self-reported questionnaires in measuring exercise intensity, duration and interval time, this study adopted a simplified criterion: weekly physical activity was dichotomised at 150 min (e.g. running, rope skipping), without specifying exercise intensity. This threshold was chosen based on the feasibility and consistency of questionnaire-based data collection and is considered more suitable for the actual conditions of college students.

### Statistical analysis

Statistical analyses were performed using SPSS 17.0. Group differences in insomnia were assessed using independent samples *t*-tests. Pearson correlation and multiple linear regression analyses were conducted to examine the associations between insomnia, dimensions of sleep disturbances and other influencing factors. A *p*-value < 0.05 was considered statistically significant.

### Ethical considerations

Ethical clearance to conduct this study was obtained from the Biomedical Research Ethics Committee of Gannan Medical University (No. 2025438). The study was conducted according to the guidelines of the Declaration of Helsinki. Written informed consent was obtained from the participants.

## Results

### General status of insomnia among vocational college students

Participants were aged 17–26 years, with a mean age of 19.26 ± 0.88 years. Among 1993 vocational college students, the AIS total score was 5.57 ± 4.23, with 957 students (48.02%) scoring ≥ 6. Higher levels of insomnia were observed in students who were female, freshmen, dissatisfied with their major, consumed alcohol, used mobile phones before sleep, exercised less than 150 min per week, spent more than 14 h per week on gaming or watching videos, or spent less than 7 h per week studying or reading. Detailed results are shown in Table 1.

**TABLE 1:** Comparison of insomnia among vocational college students with different demographic characteristics.

Factor	AIS score (Mean ± s.d.)	*t*	*P*-value
**Gender**			
Male (*n* = 1266)	5.02 ± 4.17	−7.85	< 0.001
Female (*n* = 727)	6.54 ± 4.15		
**Academic year**			
Freshman (*n* = 355)	6.62 ± 4.81	4.65	< 0.001
Sophomore (*n* = 1637)	5.35 ± 4.06		
**Satisfaction with major**			
Yes (*n* = 1494)	5.10 ± 3.98	−8.88	< 0.001
No (*n* = 499)	7.00 ± 4.62		
**Smoking**			
Yes (*n* = 156)	6.13 ± 4.77	1.52	0.129
No (*n* = 1837)	5.53 ± 4.18		
**Alcohol consumption**			
Yes (*n* = 260)	6.64 ± 4.54	4.12	< 0.001
No (*n* = 1733)	5.41 ± 4.16		
**Mobile phone use before sleep**			
Yes (*n* = 1724)	5.79 ± 4.10	5.33	< 0.001
No (*n* = 269)	4.17 ± 4.74		
**Weekly physical exercise ≥ 150 min**			
Yes (*n* = 658)	4.67 ± 4.26	−6.78	< 0.001
No (*n* = 1335)	6.02 ± 4.14		
**Weekly gaming/video watching ≥ 14 h**			
Yes (*n* = 1246)	5.92 ± 4.24	4.71	< 0.001
No (*n* = 747)	5.00 ± 4.14		
**Weekly study/reading ≥ 7 h**			
Yes (*n* = 586)	4.55 ± 4.24	−7.09	< 0.001
No (*n* = 1407)	6.00 ± 4.15		

AIS, Athens insomnia scale; s.d., standard deviation.

### General status of sleep disturbance among vocational college students

Among the 1993 vocational college students, the SDQ total score was 25.50 ± 9.46. The subscale scores were as follows: sleep restlessness or agitation (12.18 ± 5.03), mental overactivity (6.20 ± 2.57), preoccupation with the consequences of insomnia (5.11 ± 2.11) and insufficient sleep preparation (2.00 ± 1.05).

### Correlation between insomnia and dimensions of sleep disturbance

The total AIS score was significantly and positively correlated with all SDQ subscale scores among the 1993 students. Detailed results are presented in Table 2.

**TABLE 2:** Correlation between insomnia and dimensions of sleep disturbance among vocational college students (*n* = 1993).

Factor	Sleep restlessness/agitation	Mental overactivity	Preoccupation with the consequences of insomnia	Insufficient sleep preparation
AIS total score	0.62**	0.63**	0.54**	0.42**

AIS, Athens Insomnia Scale.

** , *p* < 0.01.

### Regression analysis of insomnia, sleep disturbances and related factors

To further explore the relationship between insomnia and sleep disturbances, as well as related factors among vocational college students, the total AIS score was used as the dependent variable, with SDQ subscale scores and statistically significant demographic variables as independent variables. The independent variables were coded as follows: gender (male = 1, female = 0); academic year (sophomore = 1, freshman = 0); satisfaction with major (yes = 1, no = 0); alcohol consumption (yes = 1, no = 0); mobile phone use before sleep (yes = 1, no = 0); weekly physical exercise (≥ 150 min = 1, < 150 min = 0); weekly use of mobile phone or computer for gaming or video watching (≥ 14 h = 1, < 14 h = 0); and weekly study/reading time (≥ 7 h = 1, < 7 h = 0). Multivariate linear regression analysis was performed using a forward selection approach, with variables entered stepwise based on a significance level of *P* < 0.05 to identify significant predictors. Results showed that the SDQ subscales of sleep restlessness/agitation, mental overactivity and preoccupation with insomnia consequences positively predicted insomnia severity. Additionally, female gender, freshman, dissatisfaction with one’s major, alcohol consumption and less than 7 h of weekly study/reading were associated with more severe insomnia (*F* = 160.086, *p* < 0.001). Detailed results are presented in Table 3.

**TABLE 3:** Multivariate linear regression analysis of factors affecting insomnia among vocational college students.

Factors	*B*	s.e.	*β*	*t*	*P*-value	95% CI
Constant	0.99	0.46	-	2.15	0.032	0.087–1.892
Sleep Restlessness/agitation	0.19	0.03	0.23	7.47	< 0.001	0.140–0.239
Mental overactivity	0.48	0.05	0.29	9.67	< 0.001	0.383–0.577
Preoccupation with the consequences of insomnia	0.41	0.04	0.21	9.51	< 0.001	0.327–0.496
Insufficient sleep preparation	−0.02	0.09	−0.01	−0.22	0.825	−0.187–0.149
Gender	−0.54	0.15	−0.06	−3.58	< 0.001	−0.839–0.245
Academic year	−1.04	0.18	−0.09	−5.78	< 0.001	−1.394–0.688
Satisfaction with major	−0.76	0.16	−0.08	−4.73	< 0.001	−1.081–0.447
Alcohol consumption	0.55	0.21	0.04	2.65	0.008	0.144–0.957
Mobile phone use before sleep	−0.11	0.22	−0.01	−0.53	0.598	−0.535–0.308
Weekly physical exercise	0.04	0.16	0.01	0.26	0.797	−0.274–0.357
Weekly gaming/video watching	0.28	0.15	0.03	1.88	0.060	−0.012–0.565
Weekly study/reading	−0.38	0.16	−0.04	−2.33	0.020	−0.695–0.060

Note: ∆ *R*^2^ = 0.476.

s.e., standard error; CI, confidence interval.

## Discussion

In this study, nearly 50% of participants reported experiencing insomnia in the past month. Although AIS is less commonly used in China, the detection rate of sleep problems in this study was notably higher than those reported in previous studies using the PSQI – such as Zang et al.’s finding that 17.47% of university students had poor sleep quality,^7^ and Hou et al.’s report of approximately 20% among college students from single-parent families.^29^ This may be related to the broader cultural context in China, where a strong emphasis on excellence often leads vocational college students to be viewed as less capable compared with their university counterparts.^30^ Previous studies have reported that vocational students frequently experience a stronger sense of being undervalued, which is closely associated with negative emotions, learning burnout and mobile phone addiction.^31^ When students internalise this perceived bias toward their academic performance or prospects, they may experience heightened anxiety and increased rumination at night.^32^ These psychological stressors may interfere with sleep initiation and sleep quality, thereby increasing the risk of insomnia among vocational college students.^33^

This study found a significant correlation between insomnia and sleep disturbances. Specifically, sleep restlessness or agitation, mental overactivity and preoccupation with the consequences of insomnia were all significant predictors of insomnia severity. Vocational college students who have difficulty relaxing their bodies, experience high tension before sleep, and worry about being unable to fall asleep often find it hard to relax before sleep, which leads to difficulty falling asleep and an increased likelihood of feeling sleep-deprived the next day.^34^ This, in turn, exacerbates their worries about sleep and intensifies their subjective experience of insomnia. Vocational college students with excessive mental activity often have an overactive mind before bedtime, making it difficult to fall asleep and negatively affecting sleep quality. Those who are overly concerned about the consequences of poor sleep are more likely to worry about their sleep quality, leading them to subjectively perceive themselves as having insomnia. This suggests the importance of maintaining good sleep regularity for an objective understanding of one’s sleep condition and correcting insomnia.

Our results also indicate that female students report more severe insomnia, consistent with previous studies using the PSQI.^35^ However, some studies have found no significant gender differences,^36^ which may be because of variations in research instruments used or sample populations. Gender differences in stress perception and response may explain the higher prevalence of insomnia among females. In a large-scale study examining stressful life events, males and females reported experiencing a similar number of events; however, females perceived these events as more stressful and were more likely to report experiencing difficulty falling asleep and frequent nighttime awakenings.^37,38^ This highlights the importance of targeted sleep education for female students to alleviate insomnia-related anxiety. Freshmen also report more severe insomnia. This finding aligns with Li et al.’s finding that students in the lower grades had an increased risk of poor sleep quality.^39^ One possible explanation is that freshmen are often exposed to multiple transitions at the start of college – adapting to a new learning environment, increased autonomy, irregular schedules and social adjustment pressures – which can lead to greater psychological stress, sleep schedule instability and poor sleep hygiene.^40^

In contrast, by the second year, students might have gradually adjusted to college life: academic and daily schedules become more stable, stress related to transition may decrease and sleep routines become more regular – which could explain the lower insomnia prevalence in sophomores. Moreover, students dissatisfied with their academic major were also more prone to pre-sleep anxiety, resulting in heightened arousal and impaired sleep quality. Alcohol consumption, known to negatively affect sleep,^41^ was associated with more severe insomnia. In contrast, students who engaged in post-class study and reading were more likely to develop regular routines, which may reduce the adverse effects of sleep disturbances.

Moreover, we found no significant association between physical activity and insomnia. This result contrasts with the findings of Mahfouz et al.,^42^ who reported a relationship between sleep quality and physical activity among university students in Saudi Arabia. However, it is consistent with the findings of Zhao et al.,^43^ who suggested that physical activity was not significantly linked to sleep among young adults. This may be because although some vocational college students meet the recommended weekly exercise duration, their exercise patterns are often irregular, with most physical activity concentrated on weekends. Therefore, the impact on improving sleep quality and alleviating the negative effects of insomnia remains limited. Similarly, this study also found no significant association between watching short videos before bedtime and insomnia. This finding contrasts with the results of Zhong et al.,^44^ who reported that bedtime mobile phone use negatively affects sleep. It can be suggested that although these behaviours affect sleep quality, students who use their phones for gaming or video watching often do not subjectively perceive themselves as having insomnia, resulting in a relatively minor impact on insomnia symptoms. Furthermore, the non-significant associations between physical activity, bedtime mobile phone use and insomnia may also be attributed to the use of single-item measures in this study, which may have limited the accuracy of the findings. It should be emphasised that these non-significant results are exploratory and should be viewed with caution.

### Strengths and limitations

This study has several novel contributions. Firstly, most previous research on insomnia has focused on university students, with limited attention to vocational college students. By targeting this specific population, the present study found a more severe insomnia issue compared to previous studies on university students, suggesting that sleep health among vocational college students warrants greater attention. Secondly, existing sleep quality studies in China predominantly utilise PSQI, which may not adequately capture insomnia and sleep disturbance issues. This study explored both insomnia and sleep disturbances and found a significant link between them, which may offer direction for more precise intervention planning. Thirdly, the study found that female students, those dissatisfied with their major, those with a habit of drinking alcohol and students with less than 7 h of extracurricular learning and reading time per week were more likely to experience severe insomnia. Although this study used a self-designed questionnaire with single-item measures, which imposes certain limitations, the findings still provide a valuable reference for future in-depth research on factors influencing insomnia among vocational college students. For example, the association between extracurricular learning time and insomnia warrants further investigation into specific aspects such as the timing of study sessions (e.g. studying within 1 h before bedtime) and the difficulty of study content and their effects on insomnia. Fourthly, this study enriches the empirical data on sleep health among vocational college students and provides evidence to support targeted interventions in higher education institutions.

Nevertheless, this study has several limitations. Firstly, as a cross-sectional study, it cannot establish causal relationships. Future research should consider longitudinal or interventional designs to explore causal pathways. Secondly, the data were collected through self-report questionnaires, which may be subject to response bias because of over-reporting or under-reporting, thereby affecting objectivity. Future studies should incorporate more objective measurement tools. Thirdly, as mentioned above, some variables (such as physical activity and mobile phone use) were measured using single-item questions. However, factors such as exercise intensity, timing of exercise (e.g. exercising within 20 min before bedtime),^28^ timing of phone use (e.g. using the phone to watch videos at midnight) and location of phone use (e.g. in bed versus outside of bed)^45^ may affect brain arousal and thereby influence sleep quality. Because this study only conducted a preliminary exploration, the lack of statistical significance may be influenced by many confounding factors. Scientific rigour, accuracy and generalisability are limited. Further research should expand the sample size and geographic coverage to include a broader, more diverse population across China for more representative conclusions.

### Recommendations

The findings of this study suggest that mental health education and sleep hygiene promotion in vocational colleges should be strengthened in response to the high prevalence of insomnia. Educational institutions should enhance the promotion of knowledge on sleep and insomnia, helping students develop self-management strategies.^33^ Mental health education should emphasise the importance of maintaining regular sleep routines rather than simply extending sleep duration.^46^ In addition, fostering intrinsic motivation for learning may improve sleep patterns by promoting structured routines.^47^ Health education should also remind students to avoid relying on substances such as alcohol to improve sleep, preventing a vicious cycle of insomnia.^48^ Finally, psychological counselling for students dissatisfied with their major should focus on emotional regulation and constructive approaches to study, which may indirectly improve sleep quality.^49^

## Conclusion

This study investigated the current status of insomnia, its associated factors and its relationship with sleep disturbances among students from two vocational colleges in Jiangxi Province, China. The results revealed that nearly 50% of the participants experienced insomnia in the past month. Factors associated with more severe insomnia included three dimensions from SDQ (sleep restlessness or agitation, mental overactivity and preoccupation with the consequences of insomnia). Additionally, female gender, being a freshman, dissatisfaction with one’s major, alcohol consumption and spending less than 7 h per week on extracurricular study and reading were also identified as significant risk factors. Targeted interventions addressing these factors are necessary to improve sleep quality in this population.
